# Plant Signaling Mediates Interactions Between Fall and Southern Armyworms (Lepidoptera: Noctuidae) and Their Shared Parasitoid *Cotesia icipe* (Hymenoptera: Braconidae)

**DOI:** 10.3390/insects16060580

**Published:** 2025-05-30

**Authors:** Ghislain T. Tepa-Yotto, Hilaire Kpongbe, Jeannette K. Winsou, Anette H. Agossadou, Manuele Tamò

**Affiliations:** 1 Biorisk Management Facility (BIMAF), International Institute of Tropical Agriculture (IITA-Benin), 08 BP 0932 Tri Postal, Cotonou 01000, Benin; g.tepa-yotto@cgiar.org (G.T.T.-Y.); j.winsou@gmail.com (J.K.W.); anoagossadou@gmail.com (A.H.A.); 2 Ecole de Gestion et de Production Végétale et Semencière (EGPVS), Université Nationale d’Agriculture (UNA), Kétou 02500, Benin

**Keywords:** chemical signals, *Spodoptera* spp., *Cotesia icipe*, attractant, headspace volatiles profiles, repellent

## Abstract

In Africa, the current harmful maize pest is *Spodoptera frugiperda*. Its attack can be severe and cause total economic losses. *Spodoptera eridania* is another species of the same genus, detected a few months after *S. frugiperda*’s outbreaks in West and Central Africa. Though both species share a range of host plants, socioeconomic studies are yet to provide specific figures on the potential impacts of *S. eridania*. The high and inappropriate application of insecticides to control *Spodoptera* species has negative effects on the environmental elements’ health. Semiochemical tools are increasingly exploited to design alternative pest management strategies. We hypothesize that host plants release components used by the pests and a shared parasitoid to locate the host. To verify that hypothesis, we conducted behavioral assays and GC-MS analyses to identify the potential chemical signals involved in the communications of the moths and their shared parasitoid *C. icipe*. The results showed that healthy and herbivory-induced maize and amaranth produced some chemical compounds including α-pinene, limonene, isopentyl acetate, (Z)-beta-farnesene, and methyl dodecanoate, which prospects their potential use in alternative pest management strategies for recruiting *C. icipe* to control these pests. Further work will focus on field validation to develop an alternative control strategy for the moths.

## Simple Summary

This study characterized the Spodoptera host plants volatile profiles to understand their role in FAW and SAW behaviors. A number of compounds including α-pinene, limonene, isopentyl acetate, (Z)-beta-farnesene, and methyl dodecanoate were identified as potential chemicals cues involved in the Spodoptera host plant searching behaviors. The bioactivities of those compounds in recruiting the shared FAW and SAW parasitoid C. icipe was evaluated and their potential use in alternative pest management strategies to control these pests was investigated. This study highlights the robust foundation of semiochemicals tools in developing an eco-friendly approach for controlling Spodoptera species.

## 1. Introduction

Fall armyworm (FAW) (*Spodoptera frugiperda* J.E. Smith, (1797)), (Lepidoptera: Noctuidae) and southern armyworm (*Spodoptera eridania* Stoll, (1782)) (Lepidoptera: Noctuidae) are well-known as agricultural pest species. *S. frugiperda* from America is an invasive species and was reported in West and Central Africa in 2016 [1]. *S. frugiperda* has propagated in many African and Asian countries [2], and nearly 100 countries have been invaded outside its native range [3]. Among the 350 host plants from various families reported, gramineous species have shown a high preference for *Spodoptera* species [4]. Moreover, the rapid spread of *S. frugiperda* and its extensive damage induces globally and regionally a significant problem to key staple crops (*Zea mays* L.) (Cyperales: Gramineae) and, therefore, to food security [3]. A few months after the first encounter with *S. frugiperda*, the invasive southern armyworm (SAW) pest of the same origin, *Spodotera eridania* was also reported in Western and Central Africa [5]. *S. eridania* was earlier ranked in the Americas as one of the most devastating Spodoptera pests, affecting vegetative and reproductive plant tissues of many plants including cotton, soybean, and amaranth [6,7,8]. Polyphagous status *S. eridania* may lead to substantial economic losses in various crops [9]. However, *S. eridania* has not reached the pest status in West Africa for the moment. Under current and future climates, both Spodoptera species (*S. frugiperda* and *S. eridania*) were predicted to share the same habitats in Africa with some exceptions [3,10,11].

*S. frugiperda* management by Sub-Saharan Africa (SSA) smallholder farmers mainly relies on synthetic pesticide use. The fast resistance capabilities of these pests to chemical insecticides coupled with multiple applications of high dosages or active ingredient cocktails to reduce pest populations make this method ineffective and unsustainable [12,13]. In addition, the application of high insecticide dosages is associated with–significant non-target effects killing beneficial insects–hazardous environmental pollution affecting agricultural soils and water–and with negative impact on smallholder farmers and consumers [14]. To tackle these challenges, a range of safe environmentally friendly approaches have been developed. The biological control option remains one of the most sustainable pest management approaches.

Previous studies have demonstrated that spodopterans and *Cotesia* species were highly attracted to healthy and attacked maize plant volatiles, respectively [15,16,17], but the chemical signals involved in the attraction of *S. frugiperda*, *S. eridania*, and their shared parasitoid are not known. Moreover, in agroecosystems where the two pests are present, one might expect interspecific interactions with possible volatile interference and implications on their shared host plants and parasitoid guild. The most common parasitoid species recorded on both insect hosts in Africa was *Cotesia icipe* (Hymenoptera: Braconidae) [18,19]. This study is a pioneering investigation to profile semiochemicals produced by two major host plants of both *Spodoptera* species, maize and amaranth. Therefore, we hypothesized that (i) healthy maize and amaranth plants produce compounds(s) that attract both *S. frugiperda* and *S. eridania* and (ii) the parasitoid *C. icipe* uses the volatiles from *Spodoptera* host plants to locate the pests.

## 2. Materials and Methods

### 2.1. Plants Material

The improved variety of mays (TZE) (Cyperales: Gramineae) and the local variety of amaranth (*Amaranthus cruentus* L.) (Caryophyllales: Amaranthaceae) were planted in plastic pots under a greenhouse at 25–28 °C temperature, with a relative humidity 65–70% and a photoperiod (12 dark:12 light). The soil used for plant growth was prepared by mixing 21.4 g sterilized potting medium with droppings (poultry manure) to 2.14 kg of soil. The potted experimental plants were watered daily. The amaranth seedlings from the nursery were transplanted after two weeks in plastic pots containing soil medium as described above. Two weeks after sowing maize and transplanting amaranth plants, healthy and undamaged plants were selected. The selected plants were infested by the moths for experimental purposes. Both host plants were put in separate transparent glass cages (45 × 44 × 52.5 cm) with ventilated lids covered by a muslin net (0.01 mesh) on the two lateral sides (35 × 35 cm). Mays/amaranth plant was infested by eight larvae of third instar of either *S. frugiperda* or *S. eridania* in the above cage for 12 h. Visual inspection was made to confirm that the plant organs (leaves) were chewed and successfully infested by the larvae. After 12 h of exposure, the larvae were removed, and the attacked maize and amaranth plants were used in behavioral assays.

### 2.2. Laboratory Insect Rearing

Colonies of *S. frugiperda* and *C. icipe* were established in the insectary of the Biorisk Management Facility (BIMAF) at the International Institute of Tropical Agriculture (IITA), Benin station, using feral individuals collected from farmers’ fields. *S. eridania* colonies were initiated with pupae imported from Cameroon under standard import permits from the regulatory authorities and subsequently reared as above. The insect rearing was conducted following routine mass production protocol [18]. The rearing room conditions were 25 ± 1 °C temperature, with relative humidity 70–80%, and 12 h light and 12 h dark of photoperiod. Eggs collected from female adults of *S. frugiperda* and *S. eridania* were separately incubated in 10.5 × 6 cm sterile plastic cages with ventilated lids. After three days, the emerged larvae of *S. frugiperda* and *S. eridania* were collected and put into another separate sterile plastic cage. The *S. frugiperda* and *S. eridania* larvae were fed on fresh maize sprouts, and amaranth leaves, respectively, supplied daily until pupal stage. The pupae obtained were collected and put into a new cage and kept in the same room. Emerged males and females of pests were transferred into separate transparent glass cages (45 × 44 × 52.5 cm). A piece of cotton soaked in water was dropped into the cage to humidify the cage ventilated on two sides. We used a 10% honey solution to feed the adults of *S. frugiperda* and *S. eridania*. Every day the dead insects were taken out from the cage.

The parasitoids were reared in similar facilities and conditions as described above. Twenty to thirty female adults of *C. icipe* were transferred into a cage containing 40–50 first instar larvae (less than 48 h old) of *S. frugiperda*. The insect pests were exposed to its parasitoid for 24 h. The parasitized larvae were transferred into cylindrical jars (17 × 10.5 cm) containing maize sprouts for twelve days. The feed was changed daily. The pupae obtained were transferred into new cages without feed and were monitored daily for parasitoid emergence. The emerged parasitoids were fed on the droplet of 10% honey solution until their use in experiments. The dead insects were taken out every day.

### 2.3. Olfactometer Assays

The behavioral responses of adult males and females of *S. frugiperda* and *S. eridania* to volatiles released by healthy maize and amaranth were determined using a y-tube glass of the following dimensions (stem, 16 cm; arms, 11 cm each; 80° of angle; 3.4 cm of internal diameter). Another y-tube with different dimensions (stem, 12 cm; arms, 8 cm each; 60° of angle; 1.8 cm of internal diameter) was used to evaluate *C. icipe* behavior in the same facility. The assays were conducted at 25 ± 1 °C with RH 70–80% and a photoperiod of 12:12 h (light:dark). An electronic vacuum pump (Fib Neuberger) was used to accredit the odor sources by clean air through charcoal before it entered into the y-tube. The rate of clean air in each y-tube branch was 90 mL min^−1^. The following odor treatment combinations were tested in pest behavioral assays: (1) blank vs. blank, (2) healthy maize volatiles vs. blank, (3) healthy amaranth volatiles vs. blank, (4) healthy maize volatiles vs. healthy amaranth volatiles.

For the parasitoid behavioral assays, the combinations used are the following: (1) blank vs. blank, (2) healthy maize volatiles vs. blank, (3) healthy amaranth volatiles vs. blank, (4) *S. frugiperda*-attacked maize volatiles vs. blank, (5) *S. frugiperda*-attacked amaranth volatiles vs. blank, (6) *S. eridania*-attacked maize volatiles vs. blank, (7) *S. eridania*-attacked amaranth volatiles vs. blank, (8) healthy maize volatiles vs. healthy amaranth volatiles, (9) healthy maize volatiles vs. *S. frugiperda*-attacked maize volatiles, (10) healthy maize volatiles vs. *S. eridania*-attacked maize volatiles, (11) healthy amaranth volatiles vs. *S. frugiperda*-attacked amaranth volatiles, (12) healthy amaranth volatiles vs. *S. eridania*-attacked amaranth volatiles, (13) *S. frugiperda*-attacked maize volatiles vs. *S. eridania*-attacked maize volatiles, (14) *S. frugiperda*-attacked amaranth volatiles vs. *S. eridania*-attacked amaranth volatiles, (15) *S. frugiperda*-attacked maize volatiles vs. *S. frugiperda*-attacked amaranth volatiles, (16) *S. eridania*-attacked maize volatiles vs. *S. eridania*-attacked amaranth volatiles.

Three to four potted plants (15 days of sowing/transplanting) of each host plant and both sexes of *S. frugiperda* and *S. eridania*, and female *C. icipe* were tested. The odor source was changed at the end of the experiment every day. The position of the odors and arms connected to the treatments and controls was changed after five insects were tested to avoid a bias of position. Zero parfum liquid soap and ethanol were used, respectively, to clean and rinse the y-tube and dry it for 5 min after five replicates. Every day, at the end of the experiment the used equipment was cleaned using the same procedure, rinsed with distilled water, and then with acetone and sterilized in an autoclave at 120 °C for 1 h and then dried overnight under UV hood light.

During the experiment, one of the target insects was inserted into the Y-tube through its entrance and its choice was recorded. The observation time was set at 10 min. When the introduced insect entered an arm and moved further than 6 cm inside that arm within the set-up time and spent at least 1 min, this was considered a valid choice. But if the insect got out from the selected arm, chose the second arm, and moved further than 6 cm inside that arm and spent at least 1 min, this latter was considered as its choice. In case the insect changes its choice more than once and does not spend at least 1 min inside of any of the arms, this is considered a non-choice. The pests and parasitoid experiments were conducted at two different times (9 am–4 pm; and 7–10 pm). Sixty males and eight females of each Spodoptera species were tested separately. The test was replicated sixty times.

### 2.4. Plant Odors Collection

Odors from healthy and infested improved maize variety (TZE) and the local amaranth variety (*Amaranthus cruentus*) were collected for 12 h in the behavioral and chemical ecology laboratory at IITA Benin station at 25 ± 1 °C with RH 70–80%, and a photoperiod of 12 h light and 12 h dark. Polyester oven bags of 45.72 cm × 60.96 cm (Sigma Scientific, Gainesville, FL, USA) were used for volatile collections. Zero parfum soap and acetone were used to clean and rinse, respectively, the oven bags. The cleaned bags were baked for 30 min in an autoclave at 120 °C, then dried under a fume hood for 2 h. Notably, 2 mL of hexane and dichloromethane (all at 98–99% purity) were used separately to clean porapak Q adsorbents The cleaned porapak Q were dried under a gentle stream of white spot nitrogen. The plants used here were treated in the same manner as described above. Healthy/infested plants were delicately inserted in the oven bag and the bag was tied to the stem of the plant with a rubber band. The surface of the plant’s background (soil) and the hole pot were completely covered with aluminum foil. Flow of clean air in the bag was provided by a field pump via two Teflon tubes whose one pushes air at 100 mL/min into the bag containing a plant and the second pulls volatiles via porapak Q at 80 mL/min for 12 h. Trapping has been replicated five times. The sampled Porapak Q was eluted with 200 µL dichloromethane. The sample was concentrated at 100 µL using white nitrogen. The sample obtained was either analyzed immediately or kept at −80 °C until its use.

### 2.5. Chemical Analyses

Chemical analysis was conducted using Agilent Technologies Inc., Santa Clara, CA, USA. Series B 8890 gas chromatography (GC) equipped with a flame ionization detector and fused silica capillary column HP-5 MS (30 m × 0.25 mm × 0.25 μm). The carrier gas used was helium at a flow rate of 6.7 psi. The temperature of the injector and detector was set at 280 and 290 °C, respectively. The separation and identification of headspace volatile components were conducted by coupling GC (Agilent 8890) to MS (Agilent 5977B) in the electron impact mode at 70 eV with the splitless mode. GC settings were as follows: the initial oven temperature was 35 °C for 5 min and increased gradually every minute by 10 °C (10 °C min^−1^) to 280 °C and held for 10.5 min, then 5 °C min^−1^ to 285 °C and held at this temperature for 9 min. The total run time is 50 min.

The scan range 20 to 550 *m*/*z* at 2 scans s^−1^ was used to generate the spectra. The components’ identities were determined by comparing their retention times to the standards retention times using the library (Adams2.L, Adams2.L & NIST11.L). The confirmation of the component identities was made by comparing the mass spectra and retention times index of identified components to those stored in the libraries. The retention index was calculated using the following formula:

I = 100 × [n + (N − n) (logtr(comp) − logtr(n)/logtr(N) − logtr (n))]

n = number of carbons of the shorter alkane
N = number of carbons of the longer alkane
tr(n) = adjusted retention time of the shorter alkane
tr(N) = adjusted retention time of the longer alkane
tr(comp) = adjusted retention time of the identified compound

We used the calibration curves (peak area vs. concentration) from authentic standards of identified compounds to quantify the identified components.

### 2.6. Chemicals

Dichloromethane was purchased from Acros Organics, UK (purity 99%). Limonene, α-pinene, (Z)-beta-farnesene, methyl dodecanoate, and isopentyl acetate were all purchased from Sigma Aldrich, Taufkirchen, Germany, with purity ≥ 98%.

### 2.7. Identification of Plant Signals Involved in the Attraction of Spodoptera frugiperda, Spodoptera eridania, and Cotesia icipe

The y-tube described above was used to assess the effect of some common, specific, and available key components of healthy and attacked maize and amaranth in the attraction of both sexes of *S. frugiperda*, *S. eridania,* and *C. icipe* females. Although the doses of components can also influence insect behaviors, we only used three doses of the selected components from the host plants that attracted the pests or the parasitoids compared to the repellent plant volatiles. Dichlomethane is used for the reliability of the system. The female parasitoids and pests used in bioassays were adults, aged 1–3 days. The following combinations of components from healthy plants that attracted the pest were tested: (1) Dichloromethane (DCM) vs. Dichloromethane, (2) Dichloromethane vs. Clean air, (3) limonene vs. DCM, (4) α-pinene vs. DCM, and a blend of active components limonene and (Z)-beta-farnesene at the ratio of 1:2 vs. DCM. The different combinations of specific components from healthy and infested host plants that attracted the parasitoid and tested were: (1) DCM vs. DCM, (2) Dichloromethane vs. Clean air, (3) Methyl dodecanoate vs. DCM, (4) limonene vs. DCM, (5) (Z)-beta-farnesene vs. DCM, (6) Isopentyl acetate vs. DCM, (7) α-pinene vs. DCM, and blend of attractive components Methyl dodecanoate + limonene + (Z)-beta-farnesene at the ratio of 1:14:19 vs. DCM. The naturally occurring concentration and then higher concentrations obtained by doubling the natural concentration, and lower concentrations (half the natural concentration) of the above components (Table 1) were used in behavioral assays. The blend was prepared at the same ratio mentioned above. For each concentration, 10 microliters were picked by microsyringe and applied onto pieces of filter paper (Whatman filter No1) of 2 cm × 2 cm. The impregnated paper was dried for 30 s. Control (DCM) was placed into one arm and the impregnated filter paper into the second arm of the y-tube. The treated filters, both control and test papers, were replaced after every five individuals tested.

### 2.8. Data Analyses

The *S. frugiperda*/*S. eridania* male and female responses to healthy maize and amaranth volatile against respective controls and between both host plants, were determined using Chi-square (χ^2^) tests. *C. icipe* responses to healthy and infested host plants were performed using the same test. Also, the behavioral responses of *S. frugiperda* and *S. eridania* males and females, and *C. icipe* females to the different concentrations of the synthetic compounds against the respective controls were determined using Chi-square (χ^2^) tests. In these bioassays, 30 replicates of insects to host plants and 60 replicates of insects to synthetic compounds, that correspond to respondents (n) per experiment were considered in the analysis. All statistical analyses were performed in R software version 4.04 [20] at a 5% significance level.

## 3. Results

### 3.1. Olfactometer Assays

**Figure 1:**
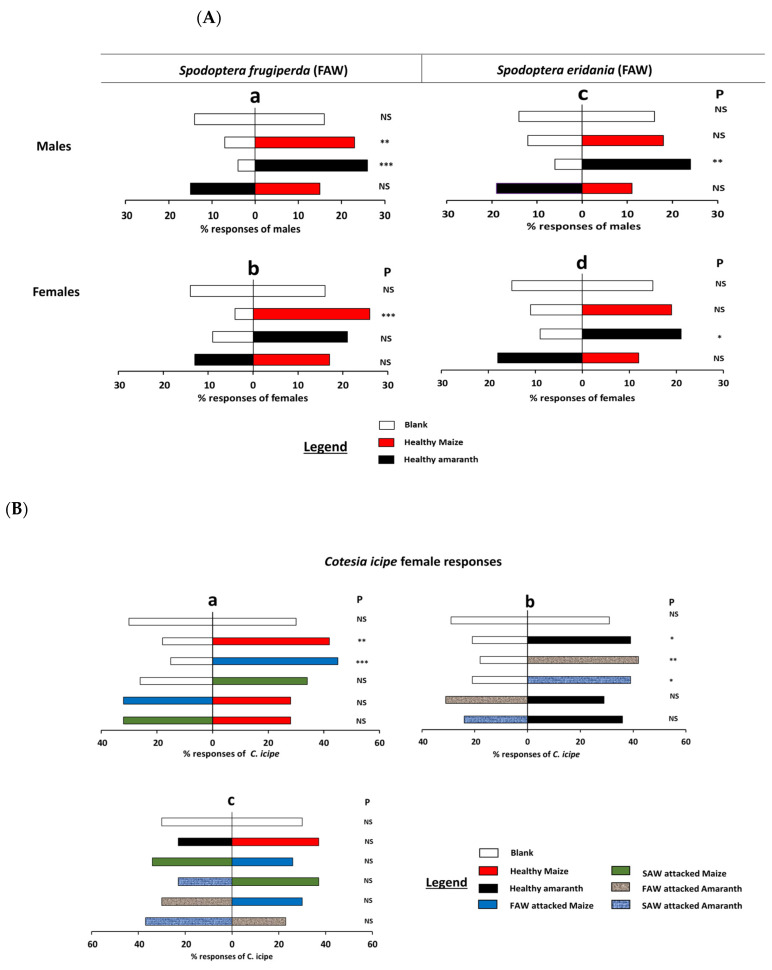
Olfactometer responses of *Spodoptera frugiperda*, *Spodoptera eridania*, and *Cotesia icipe* to maize and amaranth odors. (**A**) = *S. frugiperda*, and *S. eridania* males and female responses to headspace volatiles of healthy maize and amaranth plants. (**a**) *S. frugiperda* male responses, (**b**) *S. frugiperda* female responses, (**c**) *S. eridania* male responses, (**d**) *S. eridania* female responses. (**B**) = *Cotesia icipe* responses to headspace volatiles of healthy and infested maize and amaranth plants. (**a**) *C. icipe* female responses to maize volatiles, (**b**) C. icipe female responses to amaranth volatiles, (**c**) Choice of *C. icipe* between maize and amaranth odors. Males/females of the pests (3–4 days old), and females *C. icipe* (2–5 days old) were tested individually for a choice between blanks and odors from healthy maize and amaranth, and attacked plants. The letters a–c on the left side of Figure 2 and Figure 3 are the concentrations of each component used (Table S2A). Asterisks indicate significant difference levels: * *p* < 0.05, ** *p* < 0.01, *** *p* < 0.001. P = probability, NS = non-significant.

Male and female *S. frugiperda* and *S. eridania* did not discriminate the odor from blank and DCM, and DCM and DCM (*p* > 0.05, Table S1A). Behavioral assays of the pests showed that both sexes of *S. frugiperda* presented a strong preference for healthy maize, while the male *S. frugiperda* was only strongly attracted by healthy amaranth tested against respective control (*p* < 0.001, Table S1A). Contrariwise, no significant preference was observed when *S. frugiperda* females were exposed to healthy amaranth odor and blank (*p* = 0.02). However, *S. eridania* males and females discriminated against healthy amaranth odors when tested against control (*p* < 0.01, Table S1A). No significant difference was recorded for both sexes of *S. eridania* when they were tested against healthy maize with blank (*p* > 0.05, Table S1A). Likewise, males and females of *S. frugiperda* and *S. eridania* did not discriminate the odor from the healthy maize when combined with healthy amaranth odor (*p* > 0.05, Figure 1A(a–d), Table S1A).

Female *C. icipe* did not discriminate between blank versus blank odor sources (*p* > 0.05, Figure 1B, Table S1B), but were significantly attracted by healthy maize and *S. frugiperda*-attacked maize when tested against control (*p* < 0.01, *p* < 0.001, Figure 1B(a), Table S1B). Similar patterns were observed when *C. icipe* was tested against healthy amaranth, *S. frugiperda*-attacked amaranth, and *S. eridania*-attacked amaranth volatile with the respective control (Figure 1B(b)) (*p* < 0.05; *p* < 0.01; and *p* < 0.05, respectively, Figure 1B(b), Table S1B). Overall, in this test, *S. frugiperda*-attacked maize, and amaranth attracted more female *C. icipe* than healthy plant odors. On the other hand, no preference was observed when *C. icipe* females were provided a choice between the same species of healthy plant odors and attacked plant odors (maize and amaranth). The same results were observed when comparing healthy plant odors from both plant species, and attacked plant odors from both plant species (*p* > 0.05, Figure 1B(c), Table S1B).

### 3.2. Chemical Analysis

Chemical analysis of headspace volatile of healthy maize and amaranth showed a similarity in chemical profiles with some common components, mainly monoterpenes, aromatic hydrocarbons, esters, and aldehydes. Quantitative and qualitative differences were recorded between both profiles with specific components. Both profiles present common components, including the monoterpenes alpha-pinene and limonene (Table 2A). Furthermore, the *S. frugiperda*-attacked maize and *S. frugiperda*-attacked amaranth showed differences in chemical profiles with specific components compared to healthy plants. Some major components such as isopentyl acetate and (Z)-beta-arnesene, specific compounds for *S. frugiperda*-attacked maize were recorded (Table 2B). Methyl dodecanoate, a specific component of *S. eridania*-attacked maize, was also identified in both *S. frugiperda*-attacked amaranth and *S. eridania*-attacked amaranth (Table 2C).

### 3.3. Identification of Plant Signals Involved in the Attraction of FAW (Spodoptera frugiperda), SAW (Spodoptera eridania), and Cotesia icipe

**Figure 2:**
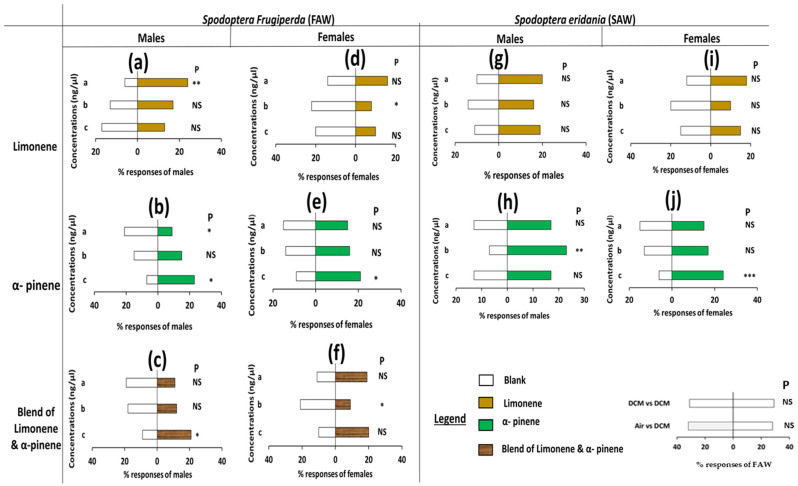
Olfactometer responses of male and female *Spodoptera frugiperda* and *Spodoptera eridania* to different concentrations of the synthetic compounds: limonene and alpha-pinene, and the blend. (**a**–**c**) responses of *S. frugiperda* males, (**d**–**f**) responses of *S. frugiperda* females, (**g**,**h**) responses of *S. eridania* males, (**i**,**j**) responses of *S. eridania* females. Thirty of each *S. frugiperda* and *S. eridania* adult males/females (3–4 days old) were tested individually for choice between three concentrations of limonene, alpha-pinene, and their blend solutions and dichloromethane. The letters a, b, and c on the left side of the figures are the concentrations of each component used. Asterisks indicate significant differences: * *p* < 0.05, ** *p* < 0.01, *** *p* < 0.001. P = probability, NS = non-significant.

Analysis of pair assay data showed that *S. frugiperda* males were significantly attracted to the lower concentration of limonene and repelled by α-pinene at lower concentrations (preferring controls) when tested against control (*p* = 0.001; *p* = 0.028, Figure 2a,c, Table S2A). Both sexes of *S. frugiperda* also showed a significant preference for the higher concentration of α-pinene and blend of α-pinene and limonene when tested against control (*p* < 0.05, Figure 2b,e, Table S2A). Likewise, females of *S. frugiperda* were significantly attracted to the natural-occurrence concentration of α-pinene when tested against respective controls (*p* = 0.05, Figure 2d,f). Contrariwise, an avoidance was observed when the *S. frugiperda* females were exposed to a choice between the natural concentration of limonene, α-pinene, a blend of these two compounds, and respective control (preferring controls) (*p* < 0.5, Table S2A). Similar results were recorded when the *S. frugiperda* female was given a choice between the low and high concentrations of limonene, a blend of limonene, α-pinene, and respective control (*p* > 0.05, Table S2A). Furthermore, no preference was recorded for both sexes of *S. eridania* to the different concentrations of limonene and the respective control (*p* > 0.05, Figure 2g,i, Table S2A). *S. eridania* males were significantly attracted to the natural concentration (*p* = 0.003, Table S2A) while the females demonstrated a significant attraction to the higher concentration (*p* = 0.001, Figure 2h,j, Table S2A).

NB: There is no significant difference when the *Spodoptera* species and the *C. icipe* were exposed to DCM vs. clean air and DCM vs. DCM (Figure 2, legend). This observation was the same for all the compounds throughout the experiments.

**Figure 3:**
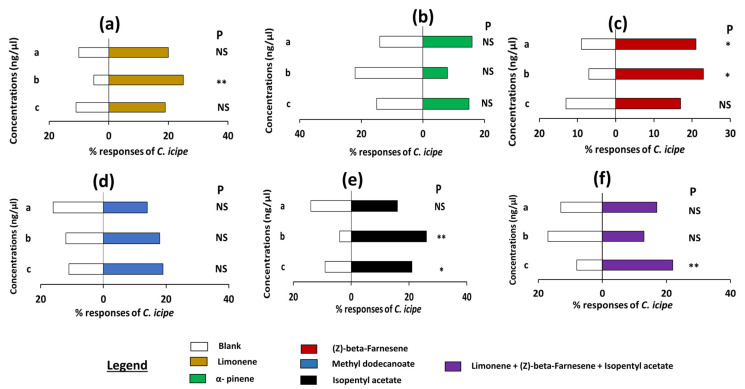
Olfactometer responses of female *C. icipe* to different doses of the synthetic compounds limonene, alpha-pinene, (Z)-beta-farnesene, methyl dodecanoate, isopentyl acetate, and the blend of the active compounds. The letters in brackets and top of each figure are the parasitoid responses to an identified volatile compound. Thirty *C. icipe* adult females (2–5 days old) were tested individually for choice between three doses of each compound solution and dichloromethane. The letters a, b, and c at the left side of the figures are the concentrations of each component used (Table 2) Asterisks indicate significant differences: * *p* < 0.05, ** *p* < 0.01, P = probability, NS = non-significant.

Interestingly, *C. icipe* females demonstrated a strong preference for the natural concentration of limonene, (Z)-beta-farnesene, and isopentyl acetate and its higher concentration when compared to the respective control (*p* < 0.01, Figure 3a,c,e, Table S2B). On the other hand, the different concentrations of methyl dodecanoate and α-pinene were not attractive to *C. icipe* females when tested against the respective control (*p* > 0.05, Figure 3b,d, Table S2B). *C. icipe* females showed a high preference for the higher concentration of the mixture of the three attractive components (limonene + (Z)-beta-farnesene + isopentyl acetate) (*p* = 0.01, Figure 3f, Table S2B).

## 4. Discussion

Our study demonstrated that both host plants maize and amaranth attracted *Spodoptera frugiperda* and *Spodoptera eridania* adult males and females. This result suggests that the maize and amaranth plants produced common chemical signals involved in recognizing and locating the host plants in the field. The herbivores utilize the plant signals to locate the food source [21] which might be the case in our study. Previous studies sustained that the host location of lepidopterous insects is chemical-based [22,23]. For example, it has been demonstrated, in the flight tunnel and field experiment, that female *S. frugiperda* exploited the blend of plant chemical compounds to locate the host [23], which corroborates our results. Moreover, our findings demonstrated that *S. frugiperda* has a high preference for maize odor compared to the amaranth plant odor, confirming previous reports [3,15,16]. Interestingly, the amaranth and maize odors attracted *S. eridania*, suggesting these host plants produce shared chemical signals that induced the attraction of both insect pests as reported previously [15]. Chemical disparities in specific compounds of healthy maize and healthy amaranth did not affect the pests (*S. frugiperda* and *S. eridania*) attractions. The findings suggest that the host plants might be interspecific hybridization sites for *S. eridania* and *S. frugiperda* in the absence of the prime host plant, potentially leading to failure risks in pest management programs if not carefully addressed [24].

The parasitoids are attracted to the volatile compounds associated with weakened or stressed host plants, and pheromone-based kairomones produced by the host insects [25,26]. The equal attraction of the solitary koinobiont larval endoparasitoid *C. icipe* for healthy maize and healthy amaranth as compared to controls suggests that no herbivory-induced volatiles might be required to call for the wasp, further indicating the possibility of pest-enemy synchrony for early parasitization before herbivory establishes and becomes devastating. The results also corroborate earlier work where *C. icipe* discriminated the maize odors when tested against companion plants [15]. The high attractiveness of *S. frugiperda*-attacked maize and *S. frugiperda*-attacked amaranth to *C. icipe* suggests that the attacked plant produced specific, herbivory-induced chemical volatiles, or higher concentrations of active healthy plant components which aligns with previous findings [15]. Our results also concur with a field study that demonstrated that *C. icipe* exploited the *Spodoptera littoralis* (Boisduval) (Lepidoptera: Noctuidae) attacked amaranth plant odor to locate and parasitize pest larvae [27]. These results suggest that the host plants produce potential attractants if identified, can be used to increase the natural enemy population in the field and thereby reduce the pest density.

GC–MS analysis detected several potential candidates as α-pinene, limonene, isopentyl acetate, (Z)-beta-farnesene, and methyl dodecanoate which are commonly associated with different plant families including Gramineae and Amaranthaceae [15,28,29]. However, their role in mediating *C. icipe* behavior in *S. frugiperda* and *S. eridania* management has not been explored yet. Furthermore, the significant changes in headspace chemical profiles of attacked maize and amaranth plants volatiles relative to healthy plants is a function of plant status as reported by a previous study [30].

The observed variability in the attraction of both sexes of *S. frugiperda* and *S. eridania* to the highest concentration as compared to the controls, naturally occurring concentration of α-pinene indicated that pest attraction is dose-dependent as reported previously [31]. Moreover, the high attraction of the pests to these concentrations suggests that this component might be used as bait in an attract-and-kill system for controlling the pest as reported in previous studies for lepidopterous insects and other insect orders [31,32,33,34]. However, in practical terms, one should consider the cost implications associated with high-concentration attractant production and use in the field.

Our findings align with previous reports [33] whereby the combination of synthetic pheromones of an ester with other components such as (Z)-3-hexenyl acetate with benzaldehyde, phenylacetaldehyde, or linalool, captured more other species of the same group as *Spodoptera exigua* Hubner, *Spodoptera littoralis* Boisduval, and *Spodoptera mauritia* Boisduval (all Lepidoptera: Noctuidae). In addition, the attraction of *S. frugiperda* males to the lowest concentration (1187 ng.uL^−1^) of limonene, suggests that males of *S. frugiperda* are more sensitive to chemical signals than females of that species, which might be the mechanism that facilitates the female location for mating, and need further investigation. Moreover, the avoidance behavior observed for female S. frugiperda to the blend of limonene and α-pinene may be an indication that the sensitivity of the insect is a function of the amounts of the compounds tested. This merits further investigation. On another note, the attraction of female *C. icipe* to the naturally occurring concentration of limonene, isopentyl acetate, and (Z)-beta-farnesene indicated that these compounds act as attractants and can be used in biological control to recruit the natural enemy in the field, which warrants further studies. Interestingly, the blend of the three experimental compounds was significantly attractive to *C. icipe*, suggesting that female *C. icipe* utilize these compounds individually or in the blend to locate the pests’ larvae for parasitism. The finding corroborates with a previous study which demonstrated the host-searching behaviors of female parasitoids of the same order: *Telenomus remus* Nixon (Hymenoptera: Scelionidae) induced by some esters such as (Z)-9-tetradecene-1-ol acetate and (Z)-9-dodecene-1-ol acetate [35].

In conclusion, we have observed that maize and amaranth plants produced chemical volatiles including α-pinene and limonene, which might be involved in spodopteran host location and need to be further assessed individually or blended for their potential in an attract-and-kill system, or in a push-pull strategy to control *S. frugiperda* and *S. eridania* populations in the field. Prominently, our study demonstrated that the chemical compounds limonene, isopentyl acetate, and (Z)-beta-farnesene associated with healthy and attacked maize and amaranth plants are potential semiochemical tools worth exploring for *C. icipe* recruitment for further biological control approaches. Another opportunity for evidence generation is whether other *S. frugiperda* and *S. eridania* parasitoids utilize the same compounds to detect the host pests. This study provides a new baseline for developing a semiochemical strategy in *S. frugiperda* and *S. eridania* management for sustainable maize production.

## Figures and Tables

**Table 1:** Different concentrations of synthetic compounds used in behavioral assays.

Concentrations (ng/μL)	Limonene	α-Pinene	(Z)-Beta-Farnesene	Methyl Dodecanoate	Isopentyl Acetate
**a**	911	415	1187	59	61
**b**	1823	831	2374	118	122
**c**	3646	1662	4748	236	244

**Table 2:** Compounds detected using GC-MS analysis of headspace volatiles of healthy and attacked maize and amaranth. (**A**) = Profiles of healthy maize and amaranth, (**B**) = Profiles of attacked maize, (**C**) = Profiles of attacked amaranth, (+) = present, (−) = absent. The compounds in bold are the available common compounds for healthy maize and amaranth and specific compounds for attacked maize and amaranth used in y-tube assays. Lit = literature, cal. = calculated, Qual = quality.

(**A**)						
Chemical Profiles of Healthy Plant Species						
RT	Library/ID	Qual	Index cal.	Index lit.	Maize	Amaranth
8.74	o-Xylene	97	847.09	855	+	+
9.28	p-Xylene	95	886.67	888	+	+
9.46	n-Nonane	81	899.87	900	+	-
**10.19**	**alpha-Pinene**	**97**	**936.00**	**931**	**+**	**+**
9.84	Anisole	64	918.81	918	-	+
10.01	Cumene	90	927.23	928	-	+
10.78	Mesitylene	90	965.44	972	+	+
11.15	Phenol	58	983.66	985	-	+
**12.06**	**Limonene**	**97**	**1032.07**	**1027**	+	+
14.97	n-Decanal	87	1209.34	1209	+	+
15.35	Benzothiazole	94	1235.65	1243	+	-
16.04	Tridecane	74	1283.45	1300	-	+
16.88	Benzene, 3-cyclohexen-1-yl-	60	1344.71	1345	+	+
17.42	Longicyclene	70	1384.59	1374	+	-
19.40	trans-Calamenene	58	1538.12	1534	+	-
20.23	1-Dodecanol, 2-hexyl-	60	1609.32	1611	-	+
21.43	Eicosane	58	1712.50	-	-	+
(**B**)						
Chemical profile of attacked maize plant						
RT	Library/ID	Qual	Index cal.	Index lit.	FAW	SAW
6.06	Phenyl ethyl alcohol	53			+	-
8.55	Ethylbenzene	91	833.06	846	-	+
8.73	o-Xylene	97	847.09	855	+	+
9.28	p-Xylene	95	886.67	888	-	+
**9.00**	**Isopentyl acetate**	**53**	**866.18**	**875**	**+**	**-**
9.83	Anisole	96	918.32	918	+	-
10.19	alpha-Pinene	97	936.14	936	+	-
11.60	n-Octanal	59	1006.66	1008	-	+
12.05	Limonene	64	1031.46	1027	+	-
12.40	1,3,6-Octatriene, 3,7-dimethyl-, (Z)-	95	1050.83	1038	+	-
13.57	4,8-Dimethyl-1,3-(Z),7-nonatriene	74	1118.18	1113	+	-
14.11	2-Ethyl hexyl acetate	53	1153.25	1159	+	-
14.97	n-Decanal	72	1209.66	1209	+	+
16.04	Tridecane	74	1283.60	1300	-	+
17.67	Tetradecane	91	1403.03	1400	+	
17.81	Dodecanal	91	1413.97	1409	-	+
**18.44**	**(Z)-beta-Farnesene**	**83**	**1461.36**	**1455**	**+**	**-**
18.62	n-Tridecanol	83	1475.15	1510	-	+
**19.25**	**Methyl dodecanoate**	**95**	**1525.66**	**1528**	**-**	**+**
(**C**)						
Chemical profile of attacked amaranth plant						
RT	Library/ID	Qual	Index cal	Index lit.	FAW	SAW
9.29	P-Xylene	95	887.49	888	+	+
11.19	alpha-Methyl styrene	91	985.42	986	+	+
11.54	(S)-3-Ethyl-4-methyl pentanol	59	1003.56	1020	+	-
12.07	Limonene	93	1032.04	1027.00	-	+
13.32	n-Undecane	56	1101.73	1100	+	-
14.91	1,3,5,7-Tetramethyl-adamantane	70	1205.48	1214	+	-
14.95	n-Decanal	87	1208.57	1209.00	-	+
15.35	Benzothiazole	96	1235.64	1228	+	-
15.63	Caprolactam	94	1254.98	1244	+	+
16.32	Tridecane	95	1303.16	1300	+	-
16.88	Benzene, 3-cyclohexen-1-yl-	97	1345.53	1345.00	-	+
**19.25**	**Methyl dodecanoate**	**91**	**1525.65**	**1528**	**+**	**-**
17.80	Dodecanal	87	1413.13	1409.00	-	+
18.63	n-Pentadecanol	87	1476.02	-	-	+
**19.26**	**Dodecanoic acid, methyl ester**	**94**	**1526.62**	**1528.00**	-	**+**
19.46	Pentadecane	74	1543.86	-	+	-
20.14	Heptadecane	91	1601.38	-	+	-
21.43	Eicosane	80	1712.50	-	-	+
25.86	Octadecanoic acid	91	2160.64	2172.00	-	+

## Data Availability

The datasets used during the current study are available at the link below: https://doi.org/10.5061/dryad.c59zw3rjh.

## Supplementary Materials

The following supporting information can be downloaded at: https://www.mdpi.com/article/10.3390/insects16060580/s1. Table S1A Statistics analyses associated with behavioral responses of FAW and SAW males and females subjected to volatiles of healthy maize and amaranth, Table S1B Statistics analyses associated to behavioral responses of *Cotesia icipe* subjected to volatiles of maize and amaranth, Table S2A *Spodoptera frugiperda* and *Spodoptera eridania* males and females behavioral responses to different concentrations of the synthetic compounds in y-tube assays, Table S2B *Cotesia icipe* females’ behavioral responses to different concentrations of the synthetic compounds in y-tube assays.
