# Supplementary material for: Plant Signaling Mediates Interactions Between Fall and Southern Armyworms (Lepidoptera: Noctuidae) and Their Shared Parasitoid Cotesia icipe (Hymenoptera: Braconidae)
Source: Insects. 2025 May 30;16(6):580. doi: 10.3390/insects16060580 (PMC12192746; doi:10.3390/insects16060580)
Supplement: Supplementary file 1 [file insects-16-00580-s001.zip › insects-3620953-supplementary.pdf]

Table S1A Statistics analyses associated with behavioral responses of FAW and SAW males and females subjected to volatiles of healthy maize and amaranth

	FAW male responses			FAW female responses			SAW male responses			SAW female responses		
combinations	χ^2	*df*	*P value*	χ^2	*df*	*P value*	χ^2	*df*	*P value*	χ^2	*df*	*P value*
Blank vs Blank	0.13	1	0.715	0.13	1	0.71	0.13	1	0.71	0	1	1
Healthy Maize vs Blank	8.53	1	0.003	16.13	1	5.90e-05	1.2	1	0.27	2.13	1	0.14
Healthy Amaranth vs Blank	16.13	1	5.904e-05	4.80	1	0.02	10.8	1	0.001	4.8	1	0.02
Healthy Maize vs healthy Amaranth	0	1	1	0.53	1	0.46	2.13	1	0.14	0.53	1	0.46

FAW = fall armyworm, SAW = southern armyworm. χ^2 = Chi-square, df = Degree of freedom

Table S1B Statistics analyses associated to behavioral responses of *Cotesia icipe* subjected to volatiles of maize and amaranth

Combinations	χ^2	*df*	*P value*
Blank vs Blank	0	1	1
Healthy Maize vs Blank	9.6	1	0.001
FAW-attacked Maize vs Blank	15	1	0.0001
SAW-attacked Maize vs Blank	1.07	1	0.301
Healthy Maize vs FAW-attacked Maize	0.27	1	0.605
Healthy Maize vs SAW-attacked Maize	0.27	1	0.605

Combinations	χ^2	*df*	*P value*
Blank vs Blank	0.06	1	0.796
Healthy Amaranth vs Blank	5.4	1	0.020
FAW-attacked Amaranth vs Blank	9.6	1	0.001
SAW-attacked Amaranth vs Blank	5.4	1	0.020
Healthy Amaranth vs FAW-attacked Amaranth	0.06	1	0.796
Healthy Amaranth vs SAW-attacked Amaranth	2.4	1	0.121

Combinations	χ^2	*df*	*P value*
Blank vs Blank	1	1	1
Healthy Maize vs healthy amaranth	3.27	1	0.070
FAW-attacked maize vs FAW-attacked amaranth	1.07	1	0.301
SAW-attacked maize vs SAW-attacked Amaranth	3.27	1	0.070
FAW-attacked maize vs SAW-attacked Maize	1	1	1
FAW-attacked Amaranth vs SAW-attacked Amaranth	3.27	1	0.070

χ^2 = Chi-square, df = Degree of freedom

Table S2A *Spodoptera frugiperda* and *Spodoptera eridania* males and females behavioral responses to different concentrations of the synthetic compounds in y-tube assays.

		Fall armyworm (*Spodoptera frugiperda*)						Southern armyworm (*Spodoptera eridania*)					
		Male responses			Female responses			Male responses			Female responses		
Combinations		χ^2	df	*P value*	χ^2	df	*P value*	χ^2	df	*P value*	χ^2	df	*P value*
Limonene	DCM vs DCM	0.13	1	0.715	0.13	1	0.715						
	Concentration **a** vs DCM	10.8	1	0.001	0.13	1	0.715	3.33	1	0.067	1.2	1	0.273
	Concentration **b** vs DCM	0.53	1	0.465	6.53	1	0.010	0.13	1	0.715	3.333	1	0.067
	Concentration **c** vs DCM	0.53	1	0.465	3.33	1	0.067	2.13	1	0.144	0	1	1
α- pinene	Concentration **a** vs DCM	4.8	1	0.028	0	1	1	0.53	1	0.465	0	1	1
	Concentration **b** vs DCM	0	1	1	0.13	1	0.715	8.53	1	0.003	0.533	1	0.465
	Concentration **c** vs DCM	8.53	1	0.003	4.8	1	0.028	0.53	1	0.465	10.8	1	0.001
Blend of limonene & α- pinene	Concentration **a** vs DCM	2.13	1	0.144	2.13	1	0.144						
	Concentration **b** vs DCM	1.2	1	0.273	4.8	1	0.028						
	Concentration **c** vs DCM	4.8	1	0.028	3.33	1	0.067						

Table S2B *Cotesia icipe* females' behavioral responses to different concentrations of the synthetic compounds in y-tube assays.

Combinations	Limonene			α- pinene			(Z)-beta-Farnesene			Methyl dodecanoate			Isopentyl acetate			Blend vs blank		
	χ^2	df	*P value*	χ^2	df	*P value*	χ^2	df	*P value*	χ^2	df	*P value*	χ^2	df	*P value*	χ^2	df	*P value*
DCM vs DCM	0.13	1	0.715															
Concentration **a** vs DCM	3.33	1	0.067	0.13	1	0.715	4.8	1	0.028	0.13	1	0.715	0.13	1	0.715	0.53	1	0.465
Concentration **b** vs DCM	13.33	1	0.0002	6.53	1	0.010	8.53	1	0.003	1.2	1	0.273	16.13	1	5.9e-07	0.53	1	0.465
Concentration **c** vs DCM	2.13	1	0.144	0	1	1	0	1	1	2.13	1	0.144	4.8	1	0.028	6.53	1	0.010

χ^2 = Chi-square, df = Degree of freedom. The letters a, b, and c are the different concentrations used with (a) half of natural concentration, (b) naturally occurring concentration, (c) double of natural concentration. A = Dose responses of both males and females of *S. frugiperda* and *S. eridania*, B = Dose responses of *C. icipe* females.
